# Decreased CD8^+^CD28^+^/CD8^+^CD28^–^ T cell ratio can sensitively predict poor outcome for patients with complicated Crohn disease

**DOI:** 10.1097/MD.0000000000007247

**Published:** 2017-06-30

**Authors:** Shi-xue Dai, Hong-xiang Gu, Qian-yi Lin, Yan-kun Wu, Xiao-yan Wang, Shao-zhuo Huang, Tiao-si Xing, Min-hua Chen, Qing-fang Zhang, Zhong-wen Zheng, Wei-hong Sha

**Affiliations:** a Department of Gastroenterology, Guangdong General Hospital and Guangdong Academy of Medical Sciences, South China University of Technology; b Department of Gastroenterology, Nanfang Hospital; c The First Clinical Medical School (Nanfang Hospital), Southern Medical University, Guangzhou, Guangdong; d Department of Clinical Medicine, Zhongshan School of Medicine, Sun Yat-sen University; e Department of Anatomy & Cell Biology, Brody School of Medicine, East Carolina University, Greenville, NC, USA; f School of Public Health, Southern Medical University, Guangzhou, Guangdong, China.

**Keywords:** CD8^+^ T cells, complication, Crohn disease, immunological balance, prognosis prediction

## Abstract

Crohn disease (CD) with complications such as penetrating, stricturing, and perianal disease is called complicated CD. The aim of this study is to test the efficiency with which the CD8^+^CD28^+^/CD8^+^CD28^–^ cell balance can predict a subsequent active stage in patients with newly diagnosed complicated CD.

Seventeen patients with complicated CD and 48 CD patients with no complications were enrolled. Blood CD8^+^ T cells were tested from all of the 65 newly diagnosed CD patients upon enrollment. The potential risk factors were compared between the 2 groups. A 30-week follow-up was performed, and the efficiency of the CD8^+^ cell balance at predicting active CD was analyzed using receiver-operating characteristic curves. The cumulative remission lasting rates (CRLRs) were analyzed using the Kaplan–Meier method.

Compared with the control CD group, patients with complicated CD were predominantly male and younger in age; they also had lower body mass indices (BMIs), higher Crohn disease activity indices (CDAIs), higher immunosuppressant and steroid prescription rates, and significantly higher surgical rates. The CD8^+^CD28^+^/CD8^+^CD28^–^ balance was associated with BMI, CDAI, steroids, and surgery. The CD8^+^CD28^+^/CD8^+^CD28^–^ ratios were significantly lower at week 0 and on the 6th, 22nd, and 30th week during follow-up with a shorter lasting time of remission for the complicated CD patients. The CD8^+^CD28^+^/CD8^+^CD28^–^ ratio could accurately predict the active stage for the patients with complicated CD, and the highest sensitivity (89.2%) and specificity (85.3%) were found when the ratio was 1.03. Treatment with steroids and surgery, along with a significantly lower CD8^+^CD28^+^/CD8^+^CD28^–^ ratio and lower CRLRs, was closely related to a worse outcome for the patients with complicated CD.

Patients requiring steroids and surgery experience more severe disease activity and thus a disequilibrated immunological balance, which could be the main reason for a decreased CD8^+^CD28^+^/CD8^+^CD28^–^ ratio. This ratio can sensitively predict the active stage for patients with complicated CD, and more care should be taken when this ratio is <1.03.

## 1 Introduction

Significant changes have been witnessed in the geographical distribution of inflammatory bowel disease (IBD); IBD has shifted from the developed world, with a high incidence rate, to traditionally low-incidence regions such as Asia, South America, Southern and Eastern Europe.^[1]^ The incidence rate is estimated to be 1.37 per 100,000 individuals in Asia and 3.44 per 100,000 individuals in China, which has the highest IBD incidence in Asia,^[2]^ emphasizing the need for specific health care resources in developing countries.

However, Crohn disease (CD) is more difficult to diagnose than ulcerative colitis (UC) because of CD's atypical and nonspecific clinical manifestations. Thus, many CD cases, approximately 52% in Asia, are finally diagnosed upon the appearance of parenteral complications, including penetrating, stricturing, perianal disease, intraperitoneal abscess, or other parenteral manifestations; these cases are classified as complicated CD.^[2]^ Almost all gastroenterologists attempt to find biomarkers or risk-prediction models to aid in early diagnosis and prognosis prediction for CD. However, no validated, inexpensive, or sensitive models for the prediction of risk are available for complicated CD.^[3]^ Increasing evidence shows that T cells, including CD4^+^ and CD8^+^ T cells and especially regulatory T cells (Tregs, mainly consisting of CD4^+^CD25^+^^[4]^ and CD8^+^CD28^–^ T cells^[5]^), play a primary role in the immunologic pathogenesis of IBD, and thus we believe that T cell subsets will be ideal biomarkers in prognosis prediction for complicated CD.

Owing to the core position of Tregs in IBD,^[6]^ we have focused on CD8^+^Tregs, which are less commonly investigated than CD4^+^CD25^+^Tregs. Our previous study showed that the balance of circulating CD8^+^CD28^+^ and CD8^+^CD28^–^ T cells, as well as the CD8^+^CD28^+^/CD8^+^CD28^–^ ratio, played a critical role in intestinal immunity, suggesting that a high level of blood CD8^+^CD28^+^ T cells is favorable for both patients and rats with UC.^[5,7]^ Furthermore, we hypothesized that the CD8^+^CD28^+^/CD8^+^CD28^–^ ratio plays a significant role in prognosis; thus, we arranged a follow-up for the patients with complicated CD at 30 weeks after diagnosis. We expected that the initial CD8^+^CD28^+^/CD8^+^CD28^–^ ratio could predict poor outcome with high sensitivity and specificity for the complicated CD patients. This study was conducted as follows.

## 2 Methods

### 2.1 Clinical data

Our investigation was approved by the Ethics Committee of Nanfang Hospital, Southern Medical University. The CD patients, with clinical diagnoses according to the local endoscopy, pathology, and pharmacy records,^[8]^ came from the Gastroenterology and Emergency departments of Nanfang Hospital, Southern Medical University, as well as the Department of Gastroenterology, Guangdong General Hospital and Guangdong Academy of Medical Sciences, between October 2013 to December 2016. CD patients with tumors, tuberculosis, autoimmune diseases, pregnancy,^[9]^ or poor compliance were excluded.^[10]^ Overall, 65 CD subjects were enrolled in this study, ranging in age from 12 to 61 years. The study participants included 17 complicated (the observation group) and 48 common (the control group) CD subjects, 46 males and 19 females. There were 8 cases who mainly suffered from penetrating (8/65, 12.31%), 12 from stricturing (12/65, 18.46%), and 6 from perianal diseases (6/65, 9.23%); 3 from ankylosing spondylitis (3/65, 4.62%); and 1 from uveitis (1/65, 1.54%); 7 patients experienced at least 2 of the above-mentioned 3 complications (7/65, 10.77%).

### 2.2 Risk factor comparison

We compared the internal factors,^[11]^ including sex, age, course of disease, body mass index (BMI), Crohn disease activity index (CDAI), and family history of bowel diseases, between the complicated and control CD groups. The external factors^[12]^ (mainly therapeutic factors) assessed included 5-aminosalicylic acid (5-ASA), immunosuppressants, steroids, probiotics, biological agents (BAs), and surgery after definite diagnosis.

### 2.3 Flow cytometry evaluation of CD8^+^T cells

CD8-FITC and CD28-PE antibodies (Santa Cruz Biotechnology, Dallas, TX) were applied to label specific circulated T cells.^[13]^ Peripheral blood was drawn after definite diagnosis for CD8^+^ T cell frequency testing. The antibody-labeled blood samples were washed and fixed, and then the cells were detected by flow cytometry (FCM) using a Beckman Coulter Epics XL flow cytometer and System II software (Beckman Coulter, Pasadena, CA).^[5,7]^ FCM was also used to measure the percentages and fluorescence intensities of the immunostained CD8^+^CD28^+^and CD8^+^CD28^–^ T cells in the sample. For each measurement, 20 × 10^4^cells were counted.^[6]^

### 2.4 Follow-up and prognosis prediction

A 30-week follow-up was performed and CD8^+^ T cells were evaluated at the end of week 0 (before definite CD diagnosis) and the 6th, 22nd, and 30th week after diagnosis (concomitantly with the infliximab time points regardless of whether the CD patients used infliximab) for all 65 CD patients. The quantities of CD8^+^CD28^+^T cells, CD8^+^CD28^–^ T cells, and the CD8^+^CD28^+^/CD8^+^CD28^–^ ratio were compared between the complicated and control CD patients at the above-mentioned 4 time points. The sensitivity and specificity of the initial (0 week) measurements of the CD8^+^CD28^+^ T cells, CD8^+^CD28^–^ T cells, and CD8^+^CD28^+^/CD8^+^CD28^–^ ratio in predicting the outcome were analyzed using receiver-operating characteristic (ROC) curves. The lasting time of remission (LTR) was calculated for the CD patients after they were determined to be in the active stage by both clinical manifestations and laboratory examinations. The cumulative remission lasting rates (CRLRs) under the different risk factors were evaluated after follow-up.

### 2.5 Statistical analysis

Repeated measures analysis of variance was used to compare the CD8^+^ T cells and their balance at different time points, and Spearman or Pearson analyses were performed to identify the correlations between CD8^+^ T cells and risk factors. ROC curves were used to verify the predicting model for CD8^+^ T cells and their balance, and the CRLRs were analyzed using the Kaplan-Meier method. The analysis was performed using the statistical software package SPSS 17.0, and statistical significance was defined at *P* < .05.

## 3 Results

### 3.1 Complicated and common CD present different risk factors

There was no significant difference in the course of the disease, stage, family history of bowel diseases, administration of 5-ASA, probiotics, or BA between the complicated and control CD cases (all *P* > .05). Compared with the control CD group, patients with complicated CD were more commonly male and younger in age; they also had lower BMIs, higher CDAIs, higher prescription rates for immunosuppressants and steroids, and a significant higher surgical rate (Table 1).

**Table 1:**
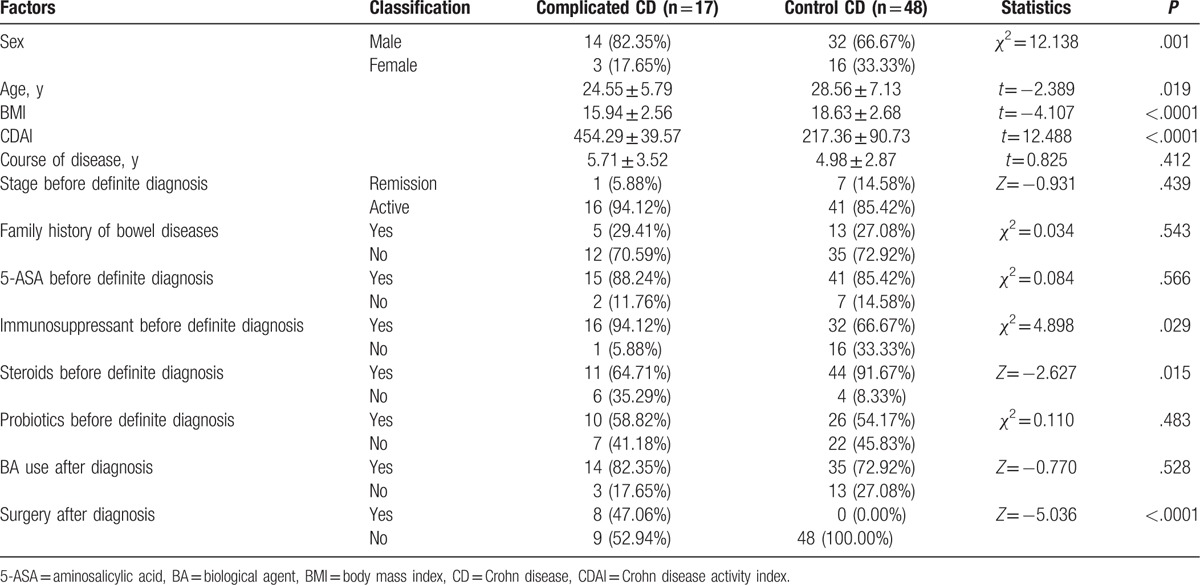
Comparison of factors between complicated and control CD (N = 65 total).

### 3.2 The CD8^+^T cell balance is correlated with risk factors

Spearman or Pearson analysis showed that the average (from week 0 to the 30th week) number of CD8^+^CD28^+^T cells and CD8^+^CD28^–^ T cells and the CD8^+^CD28^+^/CD8^+^CD28^–^ ratio were negatively correlated with CDAI, steroids, and surgery (all *P* < .05). However, only the CD8^+^CD28^+^/CD8^+^CD28^–^ ratio, but not CD8^+^CD28^+^or CD8^+^CD28^–^ T cells, was positively correlated with BMI (*P* = .001, .324 and .363, respectively; Table 2).

**Table 2:**
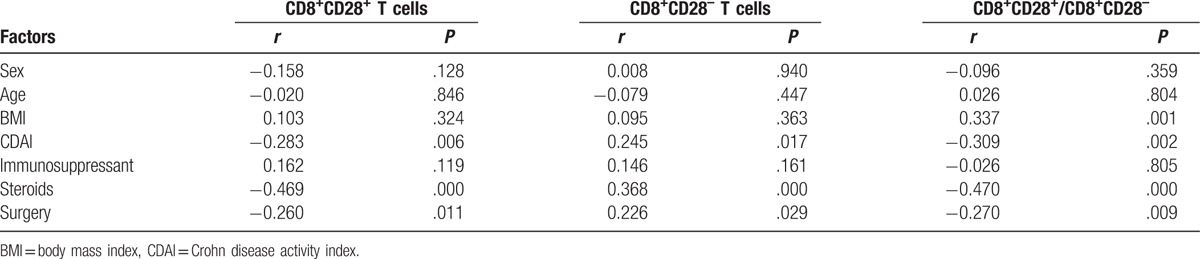
Correlation of essential risk factors with average CD8+ T cells and their balance.

### 3.3 The CD8^+^CD28^+^/CD8^+^CD28^–^ balance is increased in common CD but not in complicated CD

**Figure 1:**
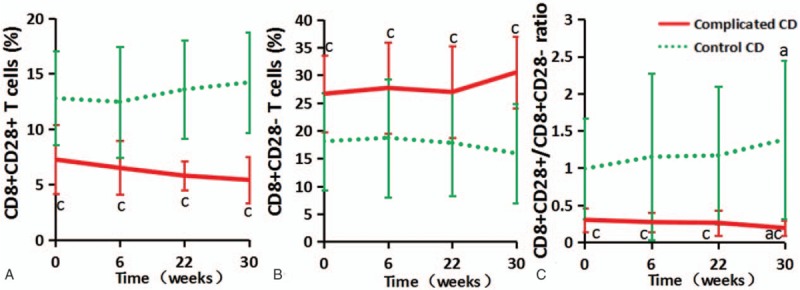
The frequencies of CD8^+^ T cells and their balance during follow-up in complicated and control CD patients. (A, B, and C depict CD8^+^CD28^+^ T cells, CD8^+^CD28^–^ T cells, and CD8^+^CD28^+^/CD8^+^CD28^–^, respectively; a represents *P* < .05 when compared to week 0, whereas c represents *P* < .05 in complicated compared to control CD patients.).

Surprisingly, the CD8^+^CD28^+^/CD8^+^CD28^–^ ratio changed in opposite directions between the complicated and control CD patients during follow-up: the ratio apparently rose with time in the control CD group but slightly decreased in the complicated CD group. At all time points (weeks 0, 6, 22, and 30), the differences in CD8^+^CD28^+^ T cells, CD8^+^CD28^–^ T cells, and CD8^+^CD28^+^/CD8^+^CD28^–^ were significant between the complicated and control CD groups (all *P* < .05, Fig. 1).

### 3.4 CD8^+^CD28^+^/CD8^+^CD28^–^ balance can effectively predict the poor outcome

**Figure 2:**
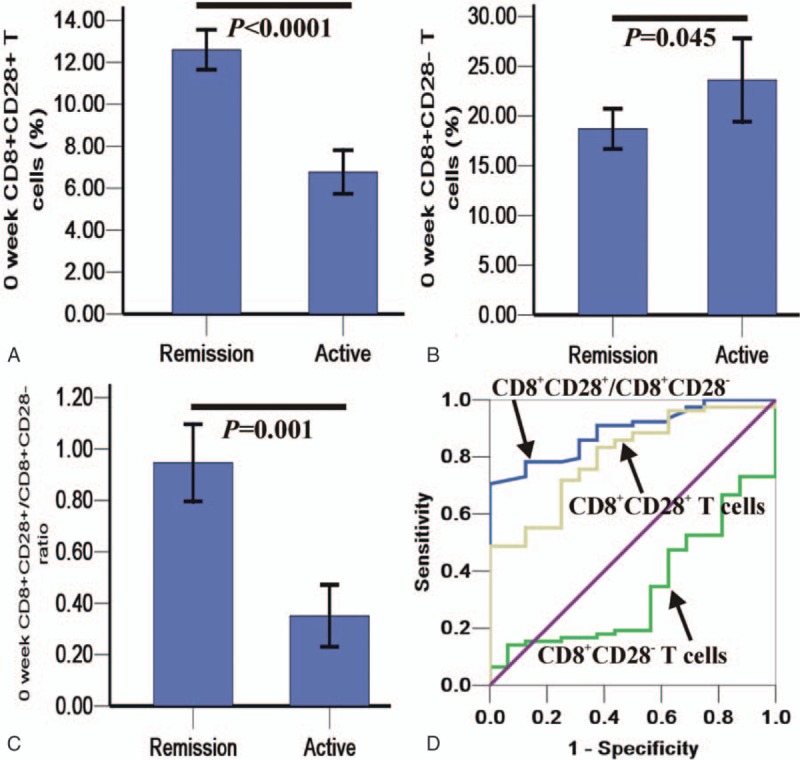
The frequencies and balance of CD8^+^ T cells in patients under remission and active Crohn disease (CD) patients before definite diagnosis (A, B, and C) and the receiver-operating characteristic (ROC) curves evaluating the ability of CD8^+^ T cells and their balance to predict the active stage in complicated and control CD patients (D). The diagonal line in D is the diagnostic reference line.

Higher initial (before definite CD diagnosis) CD8^+^CD28^+^ T cell levels and CD8^+^CD28^+^/CD8^+^CD28^–^ ratios were found in the CD group experiencing subsequent remission (n = 54, including 13 in the complicated and 41 in the control groups) when compared to the group subsequently experiencing active CD (n = 11, including 4 in the complicated group [1 case with enterovesical fistulas] and 7 in the control group; *P* < .0001 and *P* = .001; Fig. 2A and C), but the opposite was found for CD8^+^CD28^–^ T cells (*P* = .045, Fig. 2B). The LTR of the complicated group was (22.46 ± 9.02) weeks, which was significantly shorter than in the control group (30.10 ± 14.75 weeks; *P* = .044); LTR was correlated with CD8^+^CD28^+^ T cells, CD8^+^CD28^–^ T cells, and the CD8^+^CD28^+^/CD8^+^CD28^–^ ratio (*P* = .002, <.0001, and <.0001, respectively). Statistical significance was found for the initial CD8^+^CD28^+^ T cell and CD8^+^CD28^–^ T cell levels as well as the CD8^+^CD28^+^/CD8^+^CD28^–^ ratio for predicting the subsequently active stage in both complicated and control CD patients, with area under ROC curve (AUC)s of 0.802, 0.338, and 0.890, respectively (95% CIs were 0.697–0.907, 0.206–0.470, and 0.822–0.958, respectively, and *P* < .0001, *P* = .042 and *P* < .0001, respectively; Table 3 and Fig. 2D). The cutoff value showed that the best sensitivity (89.26%) and specificity (85.32%) were found when the ratio was 1.03 (9.38% CD8^+^CD28^+^ T cells /9.11% CD8^+^CD28^–^ T cells).

**Table 3:**
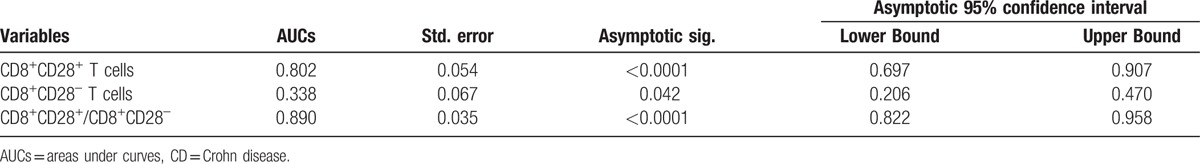
The AUCs for the ability of the initial CD8^+^ T cell levels and their balance to predict the subsequent active stage in CD patients.

### 3.5 CD patients with different risk factors had different outcomes

**Figure 3:**
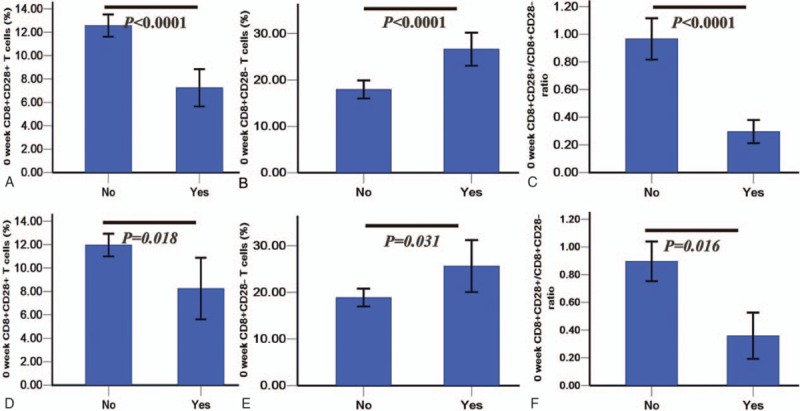
CD8^+^ T cells in nonsteroid/nonsurgery patients (No) and patients receiving steroids or undergoing surgery (Yes). A, B, and C represent comparisons between patients receiving steroids or not, whereas D, E, and F represent comparisons between patients undergoing surgery or not.

**Figure 4:**
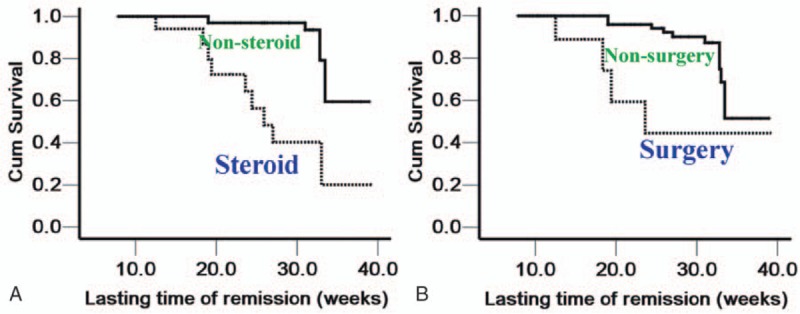
Survival plots of Crohn disease patients with or without steroid and surgery treatments. (The abscissa represents the lasting time of remission [LTR], whereas the ordinate represents the cumulative remission lasting rates. A and B depict patients receiving steroids and surgery, respectively.) (A) The median LTR of the nonsteroid group was 36.14 weeks, with a 95% confidence interval [CI] of 34.35–37.92, whereas the median LTR in the steroid group was 27.48 weeks, with a 95% CI of 23.01–31.95. B: The median LTR of the nonsurgery group was 35.09 weeks, with a 95% CI of 33.30–36.89, whereas the median LTR for the surgery group was 27.90 weeks, with a 95% CI of 20.26–35.54.

*t* Tests showed that CD8^+^CD28^+^ T cells and the CD8^+^CD28^+^/CD8^+^CD28^–^ ratio were significantly higher in the nonsteroid and nonsurgery patients than in those receiving steroids or undergoing surgery (all *P* < .05; Fig. 3A, C, D, and F), whereas the CD8^+^CD28^–^ T cell levels were significantly lower in the nonsteroid and nonsurgery patients than in those receiving steroids or undergoing surgery (*P* < .0001 and *P* = .031, respectively; Fig. 3B and E). CRLRs were significantly higher in the nonsteroid and nonsurgery CD subjects than in those receiving steroids or undergoing surgery (*χ*^2^ = 23.498 and 8.561, respectively, and *P* < .0001 and *P* = .003, respectively; Fig. 4A and B).

## 4 Discussion

### 4.1 Epidemiology and risk factors of CD complications

It has been reported that the natural history of CD includes rates of complicated disease ranging from 48% to 52% at 5 years after diagnosis.^[14]^ An Asian study^[2]^ revealed that there has been a 2- 3-fold increase in IBD incidence in several countries in Asia, and complicated and penetrating CD cases are more common in Asia than in western countries. A Chinese study^[15]^ also indicated that the clinical features of IBD in China were different from those in developed countries, regardless of age and sex distribution, disease location and severity, or the prevalence of extraintestinal manifestations. A total of 11 of 65 (16.92%) CD patients in our study were younger than 18 years, and 17 of 65 (26.15%) subjects suffered from complications, with an average disease course of 5.3 years in our study, which was not as severe as the CD cases in the United States.^[16]^

**Figure 5:**
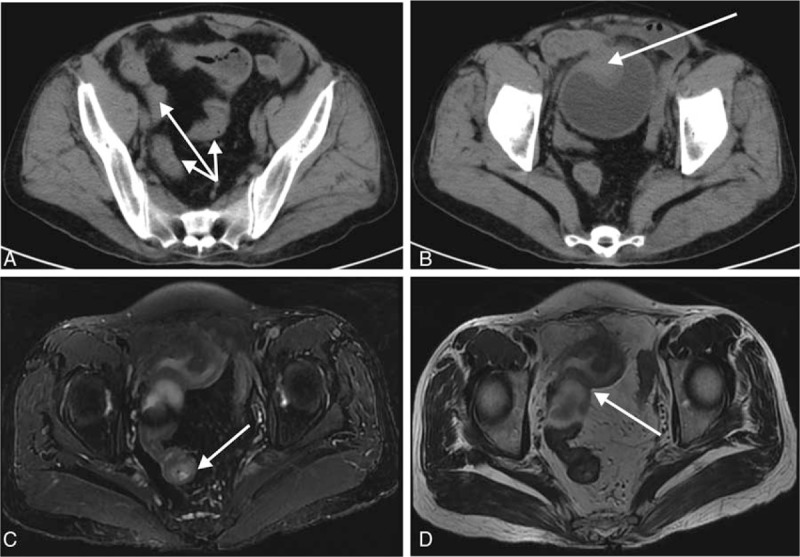
Computed tomography (CT) and magnetic resonance imaging (MRI) scanning images of a 58-year-old female with complicated Crohn disease. This patient presented with abdominal pain for >10 years, with a 3-month history of cloudy urine. Enhanced CT scanning showed a nidus with wall edema and mucosal thickness in the far end of the ileum (arrows in A), and the nidus invaded the bladder and shaped an eminence lesion, with a size of 38 × 23 mm (B). MRI scanning showed a mass convex to the enteric cavity of the ileum, with tresis and internal fistula (T2-weighted, C), and this inflammatory mass encroached on the anterosuperior bladder (T1-weighted, D).

Pathologically, stricturing generates when regeneration and repair fail to restore normal tissue architecture (as in the case of the older female with enterovesical fistulas in Fig. 5), and intestinal wall thickening can cause luminal narrowing.^[17]^ However, what clinical factors cause IBD and its complications remain unknown.^[18]^ To gain better understanding of the risk factors, we classified them into 2 types: internal and external factors. The internal factors include sex, age, age at onset, course of disease, stage, and family history, BMI, and CDAI,^[19]^ whereas the external factors often contain therapeutic factors, such as the administration of 5-ASA, immunosuppressants, steroids, BAs, and surgery.^[12,20]^ We found that no significant differences existed in the course of disease, stage, family history of bowel diseases, administration of 5-ASA, probiotics, and BA between complicated and control CD, indicating that these factors could not accelerate or retard the emergence of complications. Conversely, we found that male sex, younger age, lower BMI, higher CDAI, and higher rates of immunosuppressants, steroids and surgery were the distinguishing characteristics between the 2 groups, indicating that young and thin male CD patients are more prone to complications, consistent with Jacobsen et al's findings.^[21]^

Regarding therapeutics after definite CD diagnosis, BAs are the preferred medications if the patients have indications,^[22]^ which is the main reason why we chose weeks 0, 6, 22, and 30, corresponding to the infliximab prescription schedule, as the time points for follow-up. In our study, 49 of 65 (75.38%) subjects received infliximab, and the remaining 16 (24.62%) subjects did not undergo infliximab treatment because of financial difficulties. Unfortunately, 9 of these 16 (56.25%) cases progressed to an active stage within 30 weeks after definite diagnosis, indicating the vital therapeutic effect of BA during a short time period, which differed slightly from Lichtenstein et al's report.^[23]^ This long-term cohort study found that the mortality was similar between patients with infliximab treatments and those receiving other treatments only, whereas an increased risk of serious infection was observed with infliximab. Based on these data, we think that BAs are essential for newly diagnosed CD subjects, especially for those with complicated CD, regardless of whether they undergo surgery.

### 4.2 CD8^+^ T cells are vital in IBD follow-up

Similar to CD4^+^ T helper (Th) cells, CD8^+^ T effector (Teff) cells are the other important T lymphocyte subset that can mount both local and systemic immune responses in IBD pathogenesis.^[24]^ In mucosal immune responses, normal intestinal epithelial cells, the unique non-dendritic cell, non-monocyte, or non-B cell specialized antigen presenting cells (APCs), could express major histocompatibility complex class II molecules constitutively, expressing some critical costimulatory molecules, such as gp180,^[25]^ to activate certain peripheral blood CD8^+^ T cells selectively for a suppressor function.^[26,27]^ Furthermore, Raffaello et al^[28]^ have demonstrated for the first time that not only can circulating CD8^+^CD28^–^ suppressor T cells (Ts) be involved in the differentiation of tolerogenic APCs, but they can also suppress the Th cell response via contact mediated by APCs, implying that CD8^+^CD28^–^ suppressor T cells participate in the maintenance of peripheral tolerance. It has been confirmed that a lack of suppressor T cells (including CD8^+^CD28^–^ T cells) induced by intestinal epithelial cells is relevant to IBD.^[29]^ These data agree with Allez et al's^[30]^ finding that mucosal CD8^+^CD28^–^ T cells can function in anti-inflammatory roles, inhibiting the superabundant immunological reaction to protect the intestinal mucosa from pathological injuries, and CD8^+^CD28^+^ T cells can differentiate into CD8^+^CD28^–^ T cells to maintain active immune suppression.

Two possible mechanisms exist for how CD8^+^ T cells are involved in controlling IBD. In one mechanism, after expressing CD8α upon arrival at the intestinal epithelium, CD4^+^ T cells, with double-positive expression, could upregulate the production of interleukin (IL)-10 by suppressing Th1 on the NF-kB-GATA-3-axis.^[31]^ The other possible mechanism is that CD4^–^ CD8α^+^β^–^ T cells protect against chronic intestinal inflammation in an IL-10-dependent manner.^[32]^ Recent studies have shown that as subsets of CD8^+^ T cells, Foxp3-expressing Tregs were detected on the lamina propria (LP) of patients with IBD at a higher level than in healthy controls, whereas IL17-expressing Th17 cells were higher in patients with UC than in healthy individuals. Compared with LPMCs, both Tregs and Th17 were higher in the PBMCs of IBD patients, indicating that the plasticity in patients with IBD is led by distinct CD8^+^ T cells.^[33]^

It can be concluded from this literature that CD8^+^ T cells and their balance are vital in IBD. However, our findings seem to contradict the above-mentioned data. We found that the CD8^+^CD28^+^/CD8^+^CD28^–^ equilibrium changed inversely between complicated and control CD patients, particularly at the end of the 30th week, during follow-up: the ratio rose with time in the control CD group but declined in the complicated CD group. Moreover, at all of the time points (weeks 0, 6, 22, and 30), the CD8^+^CD28^+^/CD8^+^CD28^–^ ratios were significantly lower in complicated CD patients than in control CD patients. Surprisingly, it seemed that lower CD8^+^CD28^+^/CD8^+^CD28^–^ ratios were related to a worse outcome. The measurements were taken at the end of the 6th week during follow-up, during the expectant completion of the induced remission stage.^[20,23]^ In the control CD patients, the CD8^+^CD28^+^/CD8^+^CD28^–^ ratios increased at the end of the 22nd and 30th weeks, the time points belonging to the remission stage for most patients, which was in line with expectations. However, the ratios still decreased at these 2 time points for the complicated CD patients, indicating a more severe immunologic dysfunction caused by the complications.^[34]^ Penetrating, structuring, and anal fistula signify that more CD8^+^CD28^–^ T cells, the main force to suppress mucosal immunity, will be consumed, and more time will thus be required to increase the frequencies of CD8^+^CD28^–^ T cells, which are derived from CD8^+^CD28^+^ T cells.^[7,35]^

### 4.3 The CD8^+^CD28^+^/CD8^+^CD28^–^ ratio can effectively predict the active stage of CD

Funderburg et al^[36]^ found that the plasma levels of IL-6, interferon-α, and activated CD8^+^ T cells were increased in IBD patients compared with healthy controls, drawing an association between CD8^+^ T cell subsets and their cytokines. Although many studies were conducted to find a perfect biomarker that is helpful in the evaluation of IBD disease activity, the prognostic efficiency is not satisfactory, and an ideal prediction model has not yet been established.^[37,38]^ Based on the background, we turned to an immunological balance, the CD8^+^CD28^+^/CD8^+^CD28^–^ ratio, to validate a more powerful prediction model.

We found that a significantly lower initial CD8^+^CD28^+^/CD8^+^CD28^–^ ratio was found in the patients with subsequent active CD than in those with subsequent remission, indicating that CD relapse is associated with a decreased CD8^+^CD28^+^/CD8^+^CD28^–^ ratio. Because the lasting time of remission (LTR) can directly reflect the prognosis for IBD patients,^[39]^ and because the patients with complicated CD had a significant shorter LTR than the control subjects, we therefore performed an ROC analysis between the CD8^+^CD28^+^/CD8^+^CD28^–^ ratio and the LTR. Reassuringly, statistical significance was found for the initial CD8^+^CD28^+^ T cell count, the CD8^+^CD28^–^ T cell count, and the CD8^+^CD28^+^/CD8^+^CD28^–^ ratio for predicting the subsequent active stage for the complicated and control CD patients, verifying the effectiveness of this predictive model. The greatest efficiency was found for the CD8^+^CD28^+^/CD8^+^CD28^–^ ratio, with a sizable AUC of 0.890, which was significantly larger than CD8^+^CD28^+^ or CD8^+^CD28^–^ T cells alone. More importantly, the greatest sensitivity (89.26%) and specificity (85.32%) were found when the ratio was 1.03, indicating the key time when we should take action to treat CD patients, which is novel in the prediction model field.

Regarding the underlying mechanism for the shift in CD8^+^ T cells during follow-up, our contention is that there are 2 “shifts” in vivo that are relevant to the dynamic changes in CD8^+^ T cells and their balance: shifts from blood to colon as well as from CD8^+^CD28^+^ T cells to CD8^+^CD28^–^ T cells are crucial in the development of CD.^[30]^ In the active stage of CD, excessive mucosal immunological reactions recruit more CD8^+^CD28^–^ T cells, playing a primary role in inhibiting intestinal inflammation from the peripheral circulation to the bowel;^[31]^ thus, more circulating CD8^+^CD28^+^ T cells differentiate into CD8^+^CD28^–^ T cells to ensure a stable frequency of CD8^+^CD28^–^ T cells. The speeds of CD8^+^CD28^+^ T cell increase and CD8^+^CD28^–^ T cell decrease are not synchronous, and thus the ratio more sensitively reflects the state of immunity than do the two T cell populations alone. This hypothesis supports the phenomenon of decreased peripheral blood CD8^+^CD28^+^ T cells as well increased CD8^+^CD28^–^ T cells existing in the complicated CD patients. Based on this analysis, we speculated that the initial CD8^+^CD28^+^/CD8^+^CD28^–^ ratio could be a sensitive biomarker that can predict a poor outcome for the patients with complicated CD.

### 4.4 The CD8^+^CD28^+^/CD8^+^CD28^–^ balance is correlated with risk factors

To complete the link between immunity and clinical phenotypes,^[40]^ we performed correlation analysis on the CD8^+^CD28^+^/CD8^+^CD28^–^ ratio and the risk factors, and we found that this ratio was associated with BMI, CDAI, steroids, and surgery. It is possible that a low body weight means a poor nourishment state, and thus weakened immunity, which contributes to higher CD activation.^[41]^ Because steroid use and surgery are the main therapeutic measures and are binary variables, we subgrouped the CD patients according to undergoing steroid/surgery status.

We were somewhat surprised that the CD8^+^CD28^+^/CD8^+^CD28^–^ ratios were significantly lower in the steroid and surgery groups than in the nonsteroid and nonsurgery groups, superficially indicating that steroids and surgery are the risk factors for CD patients. Is this true? Many reports have verified that long-term and high-dose administration of steroids can significantly increase opportunistic infections in IBD patients;^[42]^ although surgery can directly remove the inflammatory foci, CD pathogenesis, and especially the underlying immunologic dysfunction, is not yet completely understood.^[43]^ Thus, some CD patients experienced recurrence after surgery, and 2 of 8 subjects in our study suffered from relapse with 30 weeks after intestinal resection (1 subject was administered prednisone at a dose of 50 mg/day before being definitively diagnosed with CD). These data suggest that steroids and surgery are double-edged swords and that the immune state is the key factor that is closely associated with recurrence.

Because the CRLRs can confirm comparisons of different risk factors, we performed a Kaplan-Meier analysis and found that CRLRs were also significantly lower in the steroid and surgery CD group when compared to the nonsteroid and nonsurgery group, which is also consistent with the comparison of the CD8^+^CD28^+^/CD8^+^CD28^–^ ratio, suggesting that the CD8^+^ T cell balance is closely related to the outcome in CD patients. As a consequence, the CD8^+^CD28^+^/CD8^+^CD28^–^ ratio is correlated not only with pathogenesis but also with the therapeutic response.

### 4.5 Conclusion and clinical significance of this study

For patients with complicated CD, depending on steroids and surgery represents more severe disease activity and thus a disequilibrated immunological balance; thus, treatment with steroids or surgery should be initiated cautiously. Based on our study, we believe that the CD8^+^CD28^+^/CD8^+^CD28^–^ balance is a novel biomarker of CD, with high sensitivity and specificity to predict the active stage for complicated CD patients. In addition, this ratio was also related to curative outcome. More clinically, when the ratio is <1.03, CD patients should follow-up more frequently so that physicians can evaluate their physical and therapeutic state to lessen the possibility of relapse. Our study has 2 main limitations: the sample size and the length of follow-up. Sixty-five subjects is a small number, and we could not avoid bias; thus, more complicated CD patients should be enrolled. A 30-week follow-up is a relatively short time period, and some long-term clinical manifestations cannot be observed during this window, especially for patients who undergo BA treatments >6 times. Moreover, how CD8^+^CD28^+^ T cells, CD8^+^CD28^–^ T cells, and the CD8^+^CD28^+^/CD8^+^CD28^–^ ratio are involved in the development of complicated CD still remains obscure and requires further investigation.

## Acknowledgments

The authors thank Professor Ye Chen, Department of Gastroenterology, and Professor Xu Li, Department of Infectious Disease, Nanfang Hospital, Southern Medical University, for their crucial suggestions in the design of this program. The authors also appreciate Xiao-juan Wu (now in the Department of Laboratory Medicine, West China Hospital, Sichuan University) and Chun-yan Wang, Department of Laboratory Medicine, Nanfang Hospital, Southern Medical University, for their dedicated work on flow cytometry for CD8^+^ T cell detection.
